# Trans-omics biomarker model improves prognostic prediction accuracy for early-stage lung adenocarcinoma

**DOI:** 10.18632/aging.102189

**Published:** 2019-08-21

**Authors:** Xuesi Dong, Ruyang Zhang, Jieyu He, Linjing Lai, Raphael N. Alolga, Sipeng Shen, Ying Zhu, Dongfang You, Lijuan Lin, Chao Chen, Yang Zhao, Weiwei Duan, Li Su, Andrea Shafer, Moran Salama, Thomas Fleischer, Maria Moksnes Bjaanæs, Anna Karlsson, Maria Planck, Rui Wang, Johan Staaf, Åslaug Helland, Manel Esteller, Yongyue Wei, Feng Chen, David C. Christiani

**Affiliations:** 1 Department of Epidemiology and Biostatistics, School of Public Health, Southeast University, Nanjing 210009, China; 2 Department of Biostatistics, Center for Global Health, School of Public Health, Nanjing Medical University, Nanjing 211166, China; 3 Department of Environmental Health, Harvard T.H. Chan School of Public Health, Boston, MA 02115, USA; 4 China International Cooperation Center for Environment and Human Health, Nanjing Medical University, Nanjing 211166, China; 5 Department of Medical Oncology, Jinling Hospital, School of Medicine, Nanjing University, Nanjing 210002, China; 6 Clinical Metabolomics Center, China Pharmaceutical University, Nanjing 211198, China; 7 Pulmonary and Critical Care Division, Department of Medicine, Massachusetts General Hospital and Harvard Medical School, Boston, MA 02114, USA; 8 Bellvitge Biomedical Research Institute and University of Barcelona, Institucio Catalana de Recerca i Estudis Avançats, Barcelona 08908, Catalonia , Spain; 9 Department of Cancer Genetics, Institute for Cancer Research, Oslo University Hospital, Oslo 0424, Norway; 10 Division of Oncology and Pathology, Department of Clinical Sciences Lund, CREATE Health Strategic Center for Translational Cancer Research, Lund University, Lund 2238, Skåne, Sweden; 11 Institute of Clinical Medicine, University of Oslo, Oslo 0424, Norway; 12 Jiangsu Key Lab of Cancer Biomarkers, Prevention and Treatment, Cancer Center, Collaborative Innovation Center for Cancer Personalized Medicine, Nanjing Medical University, Nanjing 211166, China

**Keywords:** early-stage, lung adenocarcinoma, DNA methylation, gene expression, prognostic prediction

## Abstract

Limited studies have focused on developing prognostic models with trans-omics biomarkers for early-stage lung adenocarcinoma (LUAD). We performed integrative analysis of clinical information, DNA methylation, and gene expression data using 825 early-stage LUAD patients from 5 cohorts. Ranger algorithm was used to screen prognosis-associated biomarkers, which were confirmed with a validation phase. Clinical and biomarker information was fused using an iCluster plus algorithm, which significantly distinguished patients into high- and low-mortality risk groups (*P*_discovery_ = 0.01 and *P*_validation_ = 2.71×10^-3^). Further, potential functional DNA methylation–gene expression–overall survival pathways were evaluated by causal mediation analysis. The effect of DNA methylation level on LUAD survival was significantly mediated through gene expression level. By adding DNA methylation and gene expression biomarkers to a model of only clinical data, the AUCs of the trans-omics model improved by 18.3% (to 87.2%) and 16.4% (to 85.3%) in discovery and validation phases, respectively. Further, concordance index of the nomogram was 0.81 and 0.77 in discovery and validation phases, respectively. Based on systematic review of published literatures, our model was superior to all existing models for early-stage LUAD. In summary, our trans-omics model may help physicians accurately identify patients with high mortality risk.

## INTRODUCTION

Lung cancer is the leading cause of cancer-related deaths worldwide (18.4% of total cancer deaths), with an estimated 1.76 million deaths every year [1]. Lung adenocarcinoma (LUAD) is the most common type, comprising ~40% of all cases of lung cancer, and its incidence is increasing globally [2]. With advancements in diagnostic techniques, more LUAD patients can be diagnosed at an earlier stage. Early-stage LUAD patients have a relatively superior prognosis, but even with complete surgical resection, nearly 33%–52% of patients still die from cancer within five years [3]. Molecular heterogeneity among patients might account for individual variation in LUAD survival, although the mechanism largely remains unclear [4].

Recently, great efforts have been put into using gene expression or DNA methylation data to predict the prognosis of non-small cell lung cancer (NSCLC) [5–7]. Several studies have explored prognostic prediction models for NSCLC or LUAD using molecular biomarkers from single omics data, providing opportunities to identify patients with heterogeneous prognoses [8, 9]. However, a single omics approach is insufficient to reveal the overall molecular system [10]. Accumulating evidence suggests that an integration of trans-omics features will provide comprehensive insights into multi-layered molecular mechanisms [11, 12]. Teiseira et al. [13]. Recently profiled the genomic, transcriptomic, and epigenomic landscape of prelesions of lung squamous cancer and successfully generated a predictive model to identify which lesions will progress with remarkable accuracy. However, limited prognostic models have focused on early-stage LUAD, especially with trans-omics predictors. Thus, there may be significant possibilities to develop a trans-omics prognostic model for early-stage LUAD.

Identification of molecular changes in significant oncogenes or tumor suppressor genes associated with cancer prognosis might guide early treatment and help improve survival [14]. However, most newly found genes dysregulated in cancer tissues have no effect on the neoplastic process [15]. Thus, it might be better to focus on acknowledged cancer-related genes rather than examine genes on a genome-wide scale—which is akin to looking for a needle in a haystack—to identify LUAD prognostic biomarkers. Recently, the Catalogue of Somatic Mutations in Cancer (COSMIC) identified 719 cancer-related genes through the ongoing Cancer Gene Census project. Notably, more than half of those genes participate in the development and progression of multiple tumors [16].

In this study, we hypothesized that some cancer-related genes may possess inherent potential to uncover early-stage LUAD patients with heterogeneous survival. We performed a comprehensive study of early-stage LUAD to identify prognostic-associated biomarkers from a cancer-related gene set and further accurately predicted mortality risk for patients using a trans-omics panel of clinical–DNA methylation–gene expression biomarkers.

## RESULTS

Clinical and demographic characteristics of the study population were presented in Table 1. There were 493 early-stage patients in the discovery phase (Harvard, Spain, Norway, and Sweden) and 332 early-stage patients in the validation phase (TCGA). The majority (75.2%) of LUAD patients had stage I disease. Gene expression data was available for 133 patients in Norway and 328 patients in TCGA.

**Table 1:** Baseline characteristics of the study population.

**Variables**	**Discovery phase**				**Validation phase**	**All samples (N = 825)**
	**Harvard (N = 96)**	**Spain (N = 183)**	**Norway (N = 133)**	**Sweden (N = 81)**	**TCGA (N = 332)**	
Age (years)	67.1 ± 9.9	65.6 ± 10.5	65.5 ± 9.3	66.1 ± 10.4	65.4 ± 9.8	65.7 ± 9.6
Gender, n (%)						
Female	50 (52.1)	89 (48.6)	62 (46.6)	35 (43.2)	152 (45.8)	388 (47.0)
Smoking status, n(%)						
Never	17 (17.7)	28 (15.6)	17 (12.8)	17 (21.0)	47 (14.6)	126 (15.5)
Former	52 (54.2)	97 (53.9)	74 (55.6)	39 (48.1)	194 (60.2)	456 (56.2)
Current	27 (28.1)	55 (30.6)	42 (31.6)	25 (30.9)	81 (25.2)	230 (28.3)
Clinical stage, n(%)						
I	72 (75.0)	151 (82.5)	93 (69.9)	74 (91.4)	230 (69.3)	620 (75.2)
II	24 (25.0)	32 (17.5)	40 (30.1)	7 (8.6)	102 (30.7)	205 (24.8)
Chemotherapy, n(%)						
Yes	4 (4.2)	14 (7.7)	31 (23.3)	4 (4.9)	20 (6.0)	73 (8.8)
No	92 (95.8)	142 (77.6)	102 (76.7)	50 (61.7)	109 (32.8)	495 (60.0)
Unknown	0	27	0	27	203	257
Radiotherapy, n(%)						
Yes	12 (12.5)	8 (8.9)	1 (0.8)	0 (0.0)	6 (4.7)	27 (4.8)
No	84 (87.5)	148 (91.1)	132 (99.2)	54 (100.0)	123 (95.3)	541 (95.2)
Unknown	0	27	0	27	203	257
Adjuvant therapy, n(%)						
Yes	14 (14.5)	21 (13.4)	32 (24.0)	4 (7.4)	25 (19.3)	96 (16.9)
No	82 (85.5)	135 (86.6)	101 (76.0)	50 (92.6)	104 (80.7)	472 (83.1)
Unknown	0	27	0	27	203	257
Survival year						
Median survival year	7.1	9.6	7.2	7.1	4.4	7.4
Censored rate^a^, %	0.3	58.5	68.4	40.7	80.7	63.4%

^a^ Censored rate is proportion of samples lost to follow-up or alive at end of the study.

TCGA: The Cancer Genome Atlas

### Prognosis-associated DNA methylation and gene expression probes

**Figure 1:**
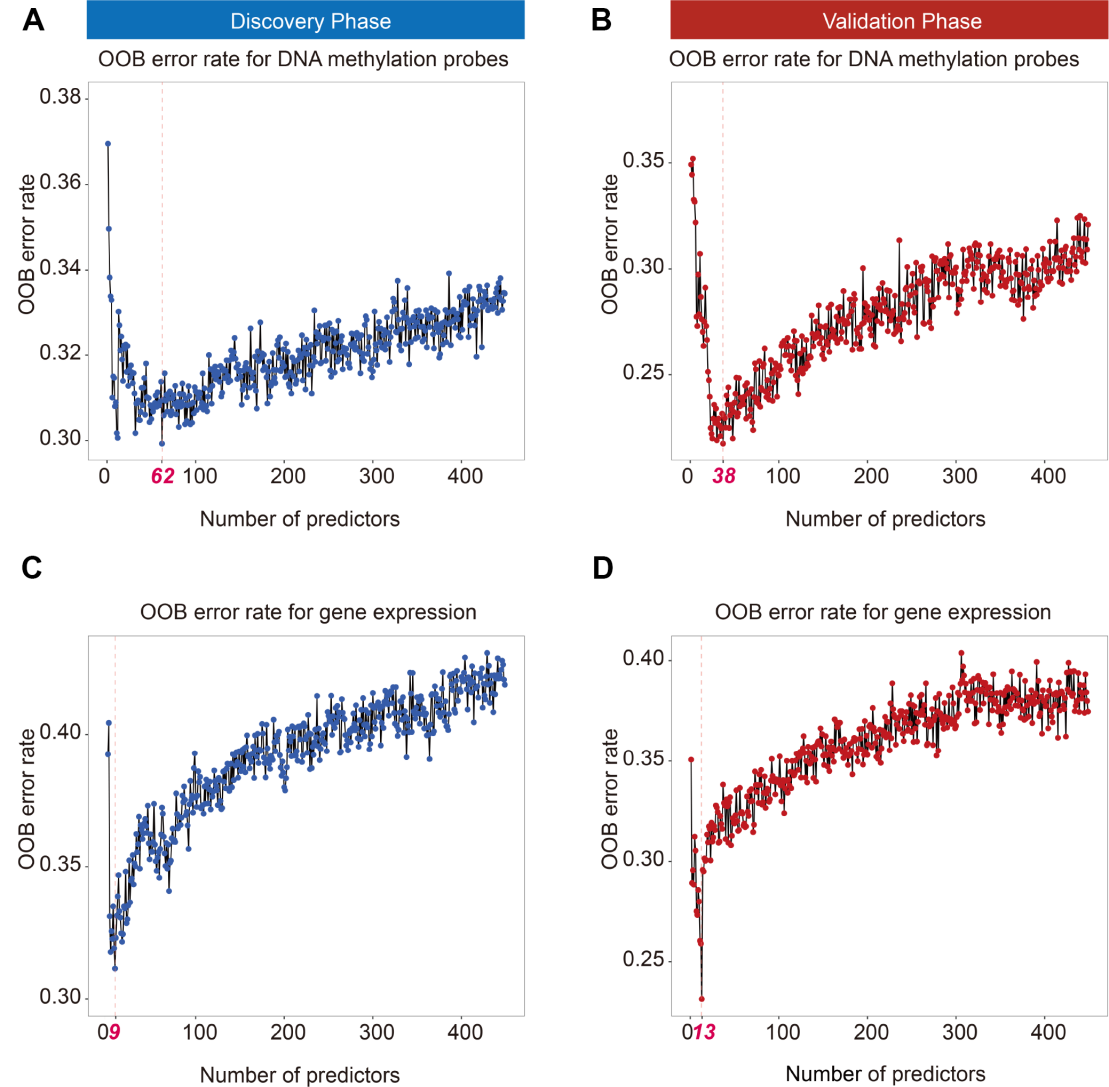
**Out of bag (OOB) error rate derived from weighted random forest analysis.** Top 62 and 38 DNA methylation probes in the discovery (**A**) and validation phases (**B**) reached a minimum OOB error rate. Top 9 and 13 mRNAs in the discovery (**C**) and validation phases (**D**) reached a minimum OOB error rate.

In total, 719 cancer-related genes from COSMIC and its corresponding 12,806 DNA methylation probes were used in this study. Ranger screened out 62 DNA methylation probes in the discovery phase according to variable importance score (VIS) (Figure 1A). Further, 38 DNA methylation probes were retained in the validation phase using the same method (Figure 1B). There were 27 overlapping DNA methylation probes between the phases, which were further analyzed by multi-Cox regression simultaneously. The 12 DNA methylation probes were significantly associated with prognosis after correction for multiple comparisons (Supplementary Table 1).

For transcriptomic analysis, the same procedure was applied to screen gene expression probes. Ranger identified 9 gene expression probes in the discovery phase (Figure 1C) and 13 gene expression probes in the validation phase (Figure 1D). Seven overlapping genes—*BLM*, *CASC5*, *FHIT*, *GMPS*, *MSH2*, *SLC34A2*, and *ZNF429*—were significantly associated with early-stage LUAD survival (Supplementary Table 2).

### Causal mediation analysis

**Figure 2:**
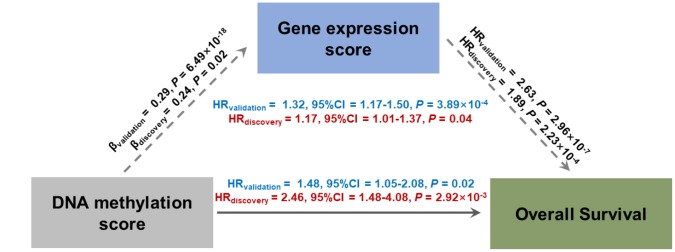
**Direct and indirect effects of DNA methylation on lung adenocarcinoma survival mediated through gene expression in casual mediation analysis.** DNA methylation risk score (MRS) and gene expression risk score (GRS) were calculated by linear combination with a weighted ln(HR_adjusted_) of identified probes.

To detect the potential mechanism by which DNA methylation affects overall survival (OS), all pairwise DNA methylation–gene expression–LUAD survival pathways were evaluated, with a consideration of *trans*- and *cis*- regulation patterns between DNA methylation and gene expression. We observed six potential causal pathways that were significant in both phases (Supplementary Table 3). Further, we calculated DNA methylation risk score (MRS) and gene expression risk score (GRS) by weighted linear combination of biomarkers using ln(HR_adjusted_) as weights. As a result, the effect of MRS on survival was significantly mediated through GRS in both phases (discovery: HR_indirect_ = 1.17, 95% CI = 1.01–1.37, *P* = 0.04, proportion mediated: 32.2%; validation: HR_indirect_ = 1.32, 95% CI = 1.17–1.50, *P* = 3.89×10^−4^, proportion mediated: 47.1%) (Figure 2).

### Patient discrimination performance of trans-omics biomarkers panel

**Figure 3:**
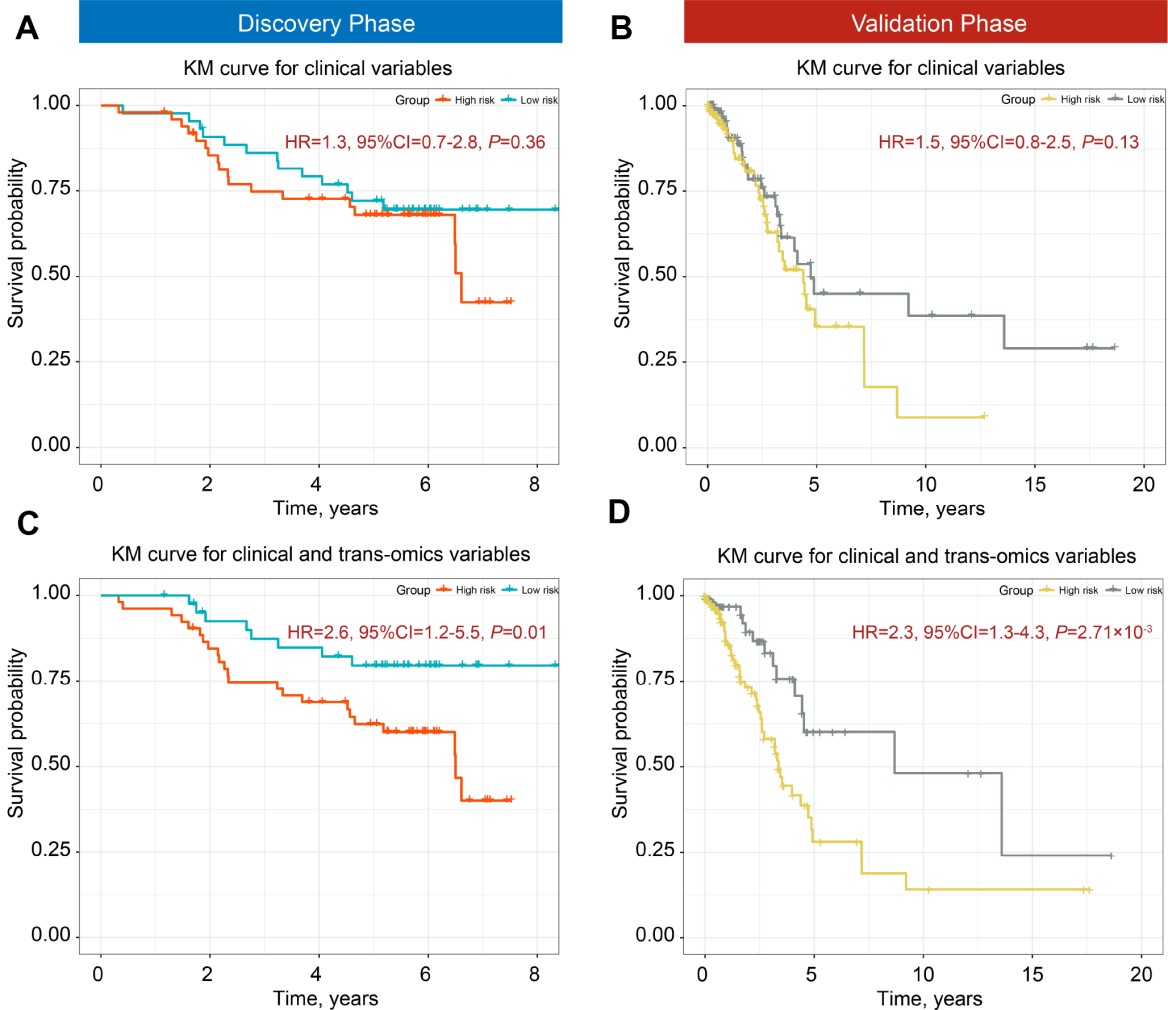
**Kaplan-Meier (KM) survial curves of high- and low-mortality risk groups divided by iCluster.** Classification ability of clinical information for discovery (**A**) and validation phases (**B**). Distinction ability of clinical information adding trans-omics biomarkers of DNA methylation and gene expression for the discovery (**C**) and validation phases (**D**).

We used the iCluster plus machine learning approach using a joint latent variable model for fusing clinical variables (age, gender, smoking status, and clinical stage) and trans-omics biomarkers (12 DNA methylation and 7 gene expression probes) to explore the classification ability of these predictors. We compared (i) clinical classifiers with (ii) clinical and trans-omics classifiers. Clinical information only was insufficient to discriminate patients into high- and low-mortality groups in both discovery and validation phases (HR_discovery_ = 1.32, 95% CI = 0.78–2.81, *P*_discovery_ = 0.363; HR_validation_ = 1.52, 95% CI = 0.86–2.53, *P*_validation_ = 0.136) (Figure 3A, 3B). However, adding DNA methylation and gene expression biomarkers resulted in significantly different survival curves between the two groups in both phases (HR_discovery_ = 2.67, 95% CI = 1.26–5.53, *P*_discovery_ = 0.011; HR_validation_ = 2.32, 95% CI = 1.32–4.31, *P*_validation_ = 2.71×10^−3^) (Figure 3C, 3D), indicating good discrimination performance of the trans-omics biomarkers panel.

### Survival prediction performance of trans-omics biomarkers panel

**Figure 4:**
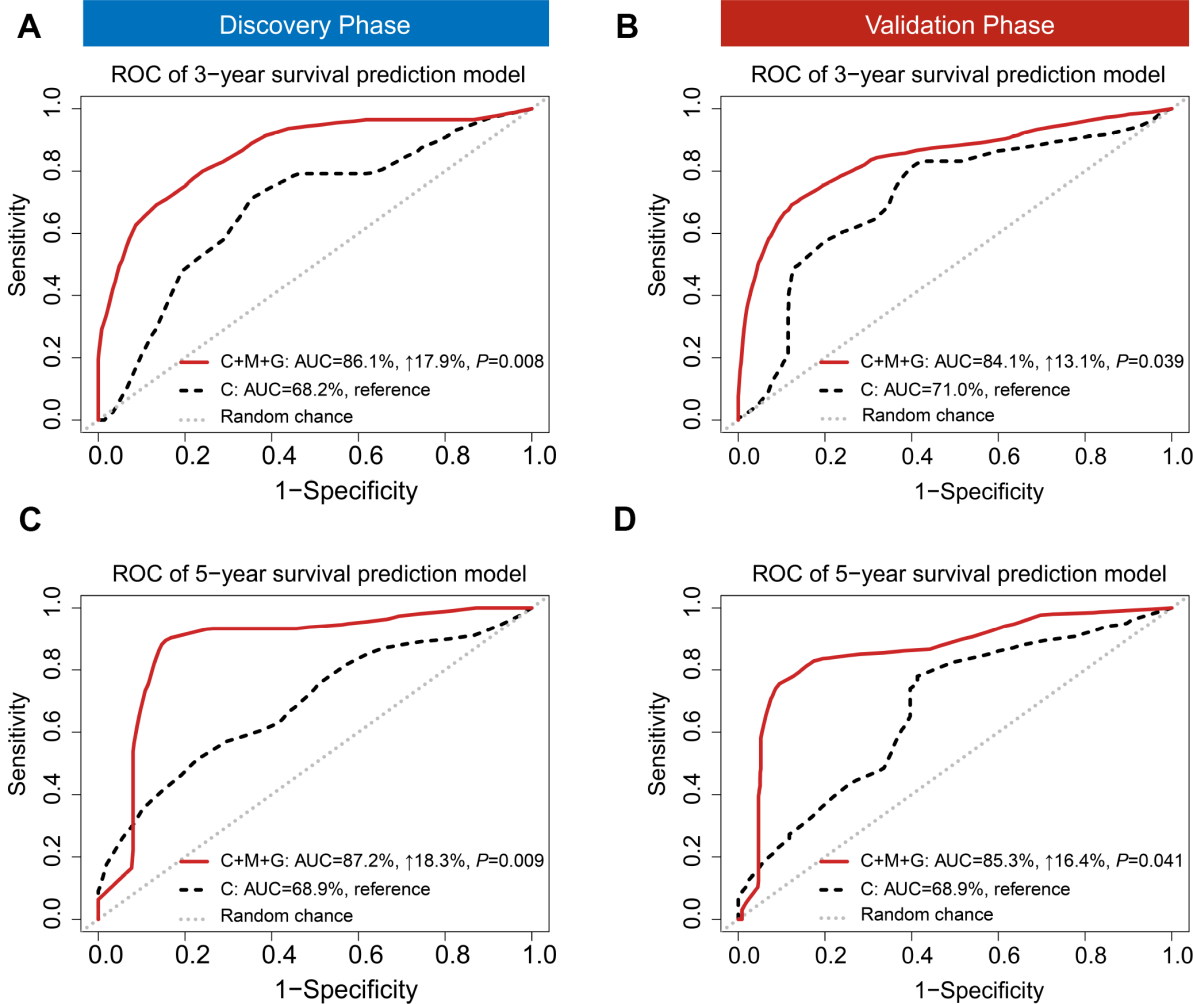
**Time-dependent receiver operating characteristic (ROC).** ROC was used to evaluate the performance of prognostic models for 3-year (**A**) and 5-year (**B**) overall survival prediction in the discovery phase. ROC also was used to evaluate the performance of prognostic models for 3-year (**C**) and 5-year (**D**) overall survival prediction in the validation phase. C: clinical model; C+M+G: clinical, DNA methylation, and gene expression model.

Besides discrimination, we used two prediction models (i) a clinical model, and (ii) a trans-omics model (clinical + MRS + GRS) to predict 3- and 5-year survival, which are the two important clinical prognostic outcomes. A risk score model was constructed with a linear combination of predictable factors weighted by the multi-Cox coefficient. Compared to the model including clinical information only, the trans-omics model significantly improved prediction accuracy in the discovery phase, with AUCs up to 86.1% for 3-year (AUC_3-year_: 17.9% increase, *P* = 0.008) (Figure 4A) and 87.2% for 5-year survival prediction (AUC_5-year_: 18.3% increase, *P* = 0.009) (Figure 4C). The validation phase further confirmed a significant improvement in prediction with the trans-omics model, with AUCs up to 84.1% for 3-year (AUC_3-year_: 13.1% increase, *P* = 0.039) (Figure 4B) and 85.3% for 5-year survival prediction (AUC_5-year_: 16.4% increase, *P* = 0.041) (Figure 4D).

### Nomogram development and validation

**Figure 5:**
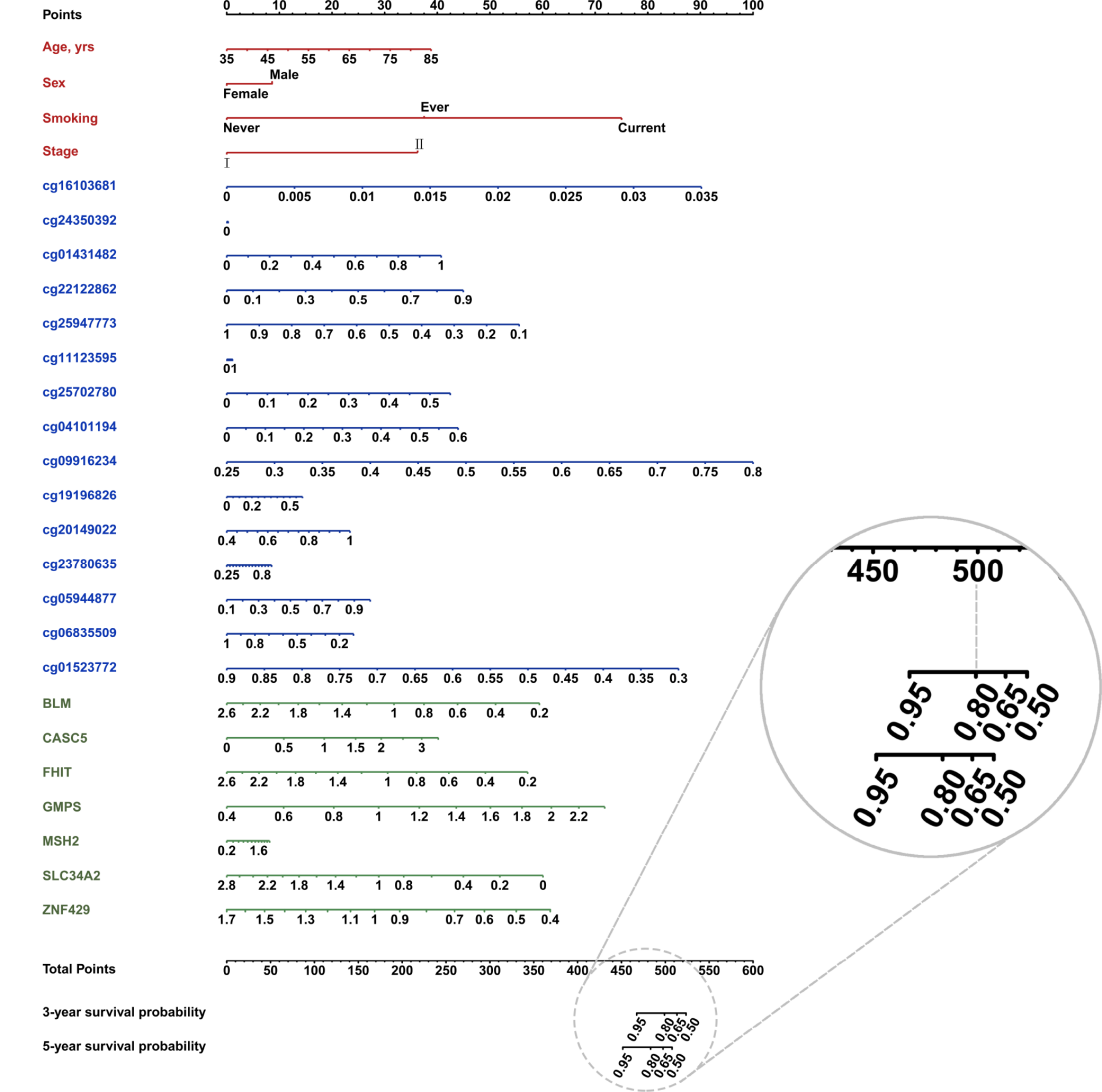
**Nomogram constructed with clinical (red font) and trans-omics biomarkers (blue and green font) for overall survival.** The probability of each predictor can be converted into the points axis in the top of the nomogram. The summary of these points of each predictor corresponded the total points at the bottom of the nomogram. After adding the points of each predictor in the total points axis, a patient’s probability of survival (3- and 5-year) can be found at the bottom of the nomogram. For example, if a patient got a score (e.g. 500), the 3-year survival probability will be corresponding to 0.80.

To easily apply our model in clinical practice, we combined clinical information and trans-omics features of patients from Norway to develop a nomogram and further test it in patients from TCGA. The nomogram was developed based on results of the multivariable Cox proportional hazards model. A weighted score calculated using all predictors was used to estimate 3- and 5-year OS (Figure 5). Discrimination and calibration methods were applied in both discovery and validation phases. c-index was calculated as 0.81 for the discovery phase (95% CI = 0.63–0.98, *P* = 6.42×10^−12^) and 0.77 for the validation phase (95% CI = 0.58–0.96, *P* = 6.80×10^−6^), indicating relatively good prediction of the nomogram. Calibration plots showed good accordance between observed OS and predicted OS for both 3- and 5-year survival in discovery and validation phases (Supplementary Figure 1).

### Sensitivity analysis

Given the potential clinical value of chemotherapy on early-stage LUAD prognosis, we further performed a sensitivity analysis to test the prediction ability of trans-omics panel using patients with available chemotherapy information. Compared to the model including clinical information only, the trans-omics model significantly improved prediction accuracy in the discovery phase, with AUCs up to 89.6% for 3-year (AUC_3-year_: 19.1% increase, *P* = 0.003) (Supplementary Figure 3A) and 90.9% for 5-year survival prediction (AUC_5-year_: 19.6% increase, *P* = 0.004) (Supplementary Figure 3C). The validation phase further confirmed a significant improvement, with AUCs up to 85.6% for 3-year (AUC_3-year_: 20.4% increase, *P* = 0.016) (Supplementary Figure 3B) and 87.2% for 5-year survival prediction (AUC_5-year_: 22.8% increase, *P* = 0.032) (Supplementary Figure 3D).

Further, we categorized all patients into two groups (age < 65 and age ≥ 65) based on the definition of elderly using UN standard [17], and evaluated whether prognostic model incorporating trans-omics biomarkers has different prediction ability between two age groups. The risk score of trans-omics biomarkers showed diverse effect on early-stage LUAD prognosis, Supplementary Figure 4 (HR_＜65_ = 2.18, 95%CI = 1.67-2.85, *P* = 5.11×10^−8^; HR_≥65_ = 3.16, 95%CI = 2.59-3.85, *P* = 3.52×10^−12^), which indicated an significant heterogeneity between the two groups (*I*^2^ = 79%, *P* = 0.03). As shown in Supplementary Figure 5, our model achieved a superior prediction performance in elderly group: AUC_≥65_ = 87.6% *v*.*s*. AUC_<65_ = 80.3% (*P* = 1.21×10^−3^) for 3-year survival prediction; AUC_≥65_ = 91.0% *v*.*s*. AUC_<65_ = 79.9% (*P* = 0.021) for 5-year survival prediction.

## DISCUSSION

Because the mechanisms underlying cancer prognosis form a complex regulatory network, integration of trans-omics data could improve prognostic value [18]. In this study, we identified 7 cancer-related genes and 12 DNA methylation probes as potentially associated with early-stage LUAD survival. By integrating clinical information and trans-omics biomarkers, we effectively classified early-stage LUAD patients into high- and low-mortality risk groups. Further, our model with a trans-omics panel of biomarkers showed preferable prediction performance for 3- and 5-year mortality.

Age is a significant risk factor of prognosis for early-stage LUAD patients, with adjustment of other covariates (HR_discovery_ = 1.02, 95% CI = 1.01-1.03, *P*_discovery_ = 0.038; HR_validation_ = 1.04, 95% CI = 1.01-1.08, *P*_validation_ = 7.35×10^−3^). Also, age was incorporated into lung cancer prognostic index in previous studies, and elderly lung cancer patients tended to have worse prognostic condition [19]. Thus, we applied our proposed model in elderly and young groups, and revealed a preferable prediction performance in elderly group for both 3- and 5-year survival, which provided insights into the exclusive benefit of clinical application for elderly early-stage LUAD patients.

To the best of our knowledge, previous studies have focused on NSCLC survival prediction, but few have examined early-stage LUAD survival prediction. However, most current prediction models are derived from clinical trials with small sample sizes [20] or single omics data [21–23]. To compare the value of our proposed model, we performed a systematic review of published literature by querying PubMed using the following search terms: “((early stage) OR (stage I) OR (stage II)) AND (lung adenocarcinoma) AND (prognosis) AND ((prediction) OR (AUC) OR (c-index))” through April 29, 2019. The complete search strategy is provided in Supplementary Figure 2.

In total, we retrieved 110 articles. After filtering by briefly screening titles and abstracts and excluding articles with irrelevant objectives or animal studies, we critically reviewed 6 potentially relevant papers. This analysis of published literature revealed that our model is more accurate than all existing models, Supplementary Table 4. Among these models, the best AUC (0.79) came from a study with only 59 patients without validation, while a study with the largest sample size (*N* = 830) and validation produced a model with low AUC (0.65). Our study sample size (*N* = 825) was comparable to the largest previous study, and we observed acceptacble AUCs (≥0.84) in both discovery and validation phases that were superior to any previous models.

Further, we calculated two indexes (AUC and c-index) to evaluate our model. Those indexes differed in numerical representation because of discrepant calculation patterns [24, 25]. For binary outcomes, the values of AUC and c-index are theoretically equivalent. However, the latter is lower for survival outcomes due to mean calculations made throughout the follow-up period. Nonetheless, the c-index calculated from our model was superior to that of previous studies.

We identified 7 genes with documented activity relevant to cancer development or prognosis or evidence of mutations in cancer that change activity of the gene product in a way that promotes oncogenic transformation. All genes discovered in this study were confirmed to participate in cancer development or prognosis. For example, *SLC34A2* produces NaPi2b, a type II sodium-phosphate cotransporter that is highly expressed on tumor surfaces of NSCLC [26]. *CASC5* interacts with high expression of *ZWINT* to lead to poor OS and disease-free survival in NSCLC [27]. Further, small cell lung tumors (80%) and NSCLC (40%) show abnormalities in RNA transcripts of *FHIT*, and 76% of the tumors exhibit loss of *FHIT* alleles [28]. *GMPs* indicate an aggressive angiogenic phenotype associated with poor prognosis in NSCLC [29]. In addition, *MSH2* is a key DNA mismatch repair protein with an important role in genomic stability, which has been confirmed to affect the risk of death in early-stage NSCLC. To our knowledge, however, there is a dearth of evidence on the role of *ZNF429* and *BLM* on the prognosis of early-stage NSCLC.

Our study identified 12 DNA methylation probes, with detailed information displayed in Supplementary Table 5. Few of their corresponding gene expression levels significantly affected OS in both discovery and validation phases. In transcript analysis, we identified 7 genes with expression robustly associated with OS. Accumulative studies indicate that an effect transmission mechanism might exist between DNA methylation probes and gene expression by *trans*-regulation patterns [30, 31]. Thus, we assumed that these 12 DNA methylation probes might affect OS partly and potentially through the 7 prognostic genes. In this way, causal mediation analysis was used to explore DNA methylation–gene expression–OS pathways. The effects of DNA methylation probes were decomposed as direct effects (affects OS independently) and indirect effects (affects OS through gene expression), and the significant indirect effects confirmed our speculation.

Our study had several advantages. (i) To our knowledge, our model is the only one with a trans-omics panel of biomarkers and achieves the best performance for early-stage LUAD survival prediction. (ii) The sample size of our study is considerably large and is comparable to the largest existing published prognostic study of early-stage LUAD. Moreover, our model also performed well in an independent population. (iii) We used two advanced statistical methods (ranger and iCluster plus) to filter out noisy biomarkers and integratively cluster patients. Ranger which can pick up molecular predictors with either main effects or interactions, is a fast implementation of random forests adjusted for covariates, and is particularly suited for signal-noise ratio enrichment in high-dimensional data analysis [32]. iCluster plus is a significant enhancement of the iCluster method, which integrates diverse data types and performs well in recognition of high- and low-mortality risk patterns [33]. (iv) Besides correction of multiple comparisons using FDR, we also used two independent phases to control false positives in biomarker testing, guaranteeing the robustness of our results.

However, we also acknowledge several limitations of our study. (i) The relationship among DNA methylation, gene expression, and OS lacks biological evidence. Thus, the association should be interpreted with caution and warrants further functional experiments. (ii) The censoring rate is high in TCGA, which may lead to low power in statistical analysis. Thus, the successfully validated biomarkers were very conservative. However, our model still achieves preferable performance, indicating its robustness. (iii) We had very limited clinical information, since several cohorts were initiated decades ago. At that time, there were few electronic records for patients. However, molecular information has significantly improved prediction performance. Adding information for laboratory tests, medical histories, and imaging examinations will improve accuracy but will also bring inconvenience for clinical application of a prediction model. Based on our results, a few easily available clinical predictors plus dozens of molecular predictors can present a balance between convenience of application and accuracy of prediction.

In conclusion, using a machine learning method and two-stage design, we built a prediction model incorporating 12 DNA methylation probes and 7 gene expression probes. These 19 molecular predictors provide perspective to design a cost-effective chip that can detect biomarkers exclusively for early-stage LAUD prognosis prediction, which will benefit both physicians and patients in clinical applications.

## METHODS

### Study population

Early-stage (stage I and II) LUAD patients (*n* = 825) were enrolled from the following five independent study centers. (1) Harvard [34]. Newly diagnosed patients with histologically confirmed primary LUAD (*n* = 96) were recruited at Massachusetts General Hospital (MGH) since 1992. Each specimen was evaluated by an MGH pathologist for amount (tumor cellularity > 70%) and quality of tumor cells and histologically classified using World Health Organization criteria. The study protocol was approved by the Institutional Review Boards at Harvard School of Public Health and MGH. (2) Spain [35]. Patients (*n* = 183) were recruited at eight sub-centers, including the Bellvitge Biomedical Research Institute (Spain), Center for Applied Medical Research (Spain), Catalan Institute of Oncology (Spain), IRCCS Foundation National Cancer Institute (Italy), University of Turin (Italy), University of Liverpool Cancer Research Centre (UK), Centre Hospitalier Universitaire A Michallon (France), and University of Michigan Medical School (USA), and the median clinical follow-up was 7.2 years. The study was approved by the Bellvitge Biomedical Research Institute Institutional Review Board. (3) Norway [36]. Patients (*n* = 133) were recruited at Oslo University Hospital-Rikshospitalet from 2006–2011. The project was approved by the Oslo University Institutional Review Board and Regional Ethics Committee (S-05307). (4) Sweden [37]. Patients (*n* = 81) were recruited at Skåne University Hospital (Lund, Sweden) from 2004–2008. Tumor DNA was collected from early-stage lung cancer patients who underwent operation at the hospital. The study was approved by the Regional Ethical Review Board in Lund, Sweden (registration no. 2004/762 and 2008/702). (5) TCGA. We also included patients (*n* = 332) from The Cancer Genome Atlas (TCGA), for which the TCGA workgroup generated level-1 HumanMethylation450 DNA methylation data (image data) and performed mRNA sequencing data processing and quality control. Datasets were downloaded on October 1, 2015.

All patients provided written informed consent under the approval of the institutional review boards of each center. Data from the international study centers were harmonized as previously described [38–41]. Quality control procedures of DNA methylation and mRNA expression data are presented in Supplementary Material.

### Statistical analysis

**Figure 6:**
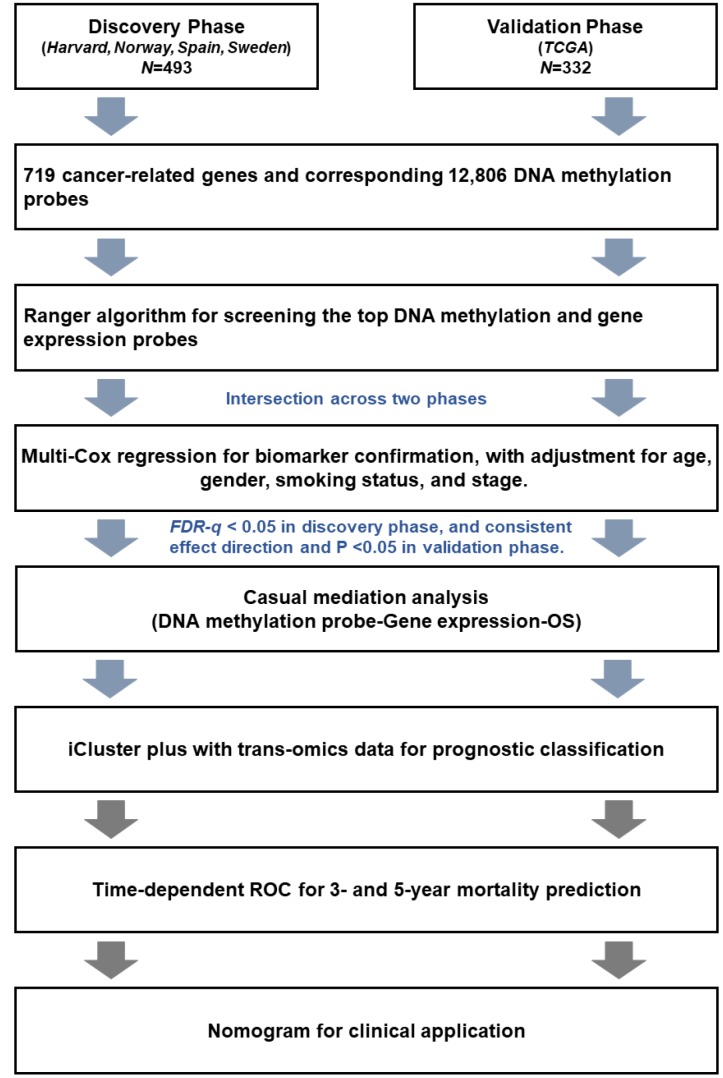
**Flow chart of the study.**

In this study, all significant results in the discovery phase were further independently confirmed in the validation phase. Patients (*n* = 493) from Harvard, Spain, Norway, and Sweden were assigned to the discovery phase. Patients (*n* = 332) from TCGA were assigned to the validation phase. The work flow chart is shown in Figure 6. First, ranger, an improved version of random forest incorporating adjustment of covariates, was used to evaluate importance of each DNA methylation probe based on VIS [32]. All DNA methylation probes were ranked by VIS in descending order. The sliding windows sequential forward feature selection method was applied to identify top important probes, which means probes were sequentially included in the model as predictors until the model reached the minimum out of bag (OOB) error rate [42]. We screened out important probes in the discovery and validation phases. Overlapping probes in both phases were retained for subsequent analysis. The same pipeline was also used to screen out important gene expression variables. Second, a multivariate Cox regression model was further used to confirm the association between each biomarker and survival with adjustment for age, gender, clinical stage, smoking status, and study center. Hazard ratio (HR) and 95% confidence interval (CI) were described per 1% methylation increment. Multiple comparison correction was performed using the false discovery rate (FDR) method by Benjamini-Hochberg procedure. Biomarkers with FDR-*q* ≤ 0.05 in the discovery phase and *P* ≤ 0.05 in the validation phase, as well as consistent effect directions across both phases, remained for further analysis. Third, we performed a causal mediation analysis to explore possible “DNA methylation → gene expression → LUAD survival” pathways [43]. Adjusting for the same covariates as above, the total effect of methylation on survival (HR_total_) was separated into indirect effects (HR_indirect_), representing the effect of methylation on survival mediated through gene expression, and direct effects (HR_direct_), representing the effect of methylation on survival directly. Fourth, we used an iCluster plus algorithm that integrated clinical and trans-omics biomarkers to distinguish patients with high- and low-mortality risk groups [33, 44]. Kaplan-Meier curves and log-rank test were used to assess survival differences between groups. Fifth, we integrated clinical and trans-omics biomarkers into a Cox regression model, and time-dependent receiver operating characteristic (ROC) curves were used to measure prediction performance for 3- and 5-year survival [45]. Area under ROC (AUC) of the prediction model with or without trans-omics biomarkers was compared using bootstrap with 1000 times resampling. Finally, nomogram plots were generated, and the validation was tested by discrimination and calibration in both phases. Discrimination was estimated by concordance index (c-index), which ranges from 0.5 (completely random) to 1.0 (perfect discrimination). Calibration assesses how close the nomogram-estimated risk is to observed risk, which was depicted by a calibration plot. Bootstrap analyses with 1000 resamples were used for these analyses. All data were analyzed using R version 3.4.4 statistical software (The R Foundation).

### Ethics approval

The Harvard study protocol was approved by the Institutional Review Boards at Harvard School of Public Health and MGH. The Spain study was approved by the Bellvitge Biomedical Research Institute Institutional Review Board. The Norway project was approved by Oslo University Institutional Review Board and Regional Ethics Committee (S-05307). The Sweden study was approved by the Regional Ethical Review Board in Lund, Sweden (registration no. 2004/762 and 2008/702). All patients provided written informed consent.

## Supplementary Material

Supplementary Methods and References

Supplementary Figures

Supplementary Tables
