# Supplementary material for: Trans-omics biomarker model improves prognostic prediction accuracy for early-stage lung adenocarcinoma
Source: Aging (Albany NY). 2019 Aug 21;11(16):6312–35. doi: 10.18632/aging.102189 (PMC6738411; doi:10.18632/aging.102189)
Supplement: Supplementary Methods and References [file aging-11-102189-s001.pdf]

SUPPLEMENTARY METHODS AND REFERENCES

Quality control procedures of DNA methylation data

DNA methylation was profiled using Infinium HumanMethylation450 BeadChips (Illumina Inc., San Diego, CA, USA) for all patients. All centers followed the same quality control procedures before the association study. Raw image data were transformed into beta values to perform background subtraction and control normalization. Unqualified probes were excluded if they met any of the following criteria: (i) detection $P > 0.05$ in more than 5% of patients; (ii) coefficient of variance < 5%; (iii) common single nucleotide polymorphisms located in probe sequence or in 10-bp flanking regions; (iv) cross-reactive probes or cross-hybridizing probes; or (v) probes not passing quality control in all centers. Samples with >5% undetectable probes were excluded. Methylation signals were further processed for quantile normalization, design bias correction for type I and II probes, and batch effects adjustment.

Quality control procedures of mRNA expression data

mRNA expression was assessed using SurePrint G3 human GE, 8 × 60 K gene expression microarrays (Agilent Technologies, Santa Clara, CA, USA) in discovery phase. TCGA mRNA sequencing data processing and quality control was done by the TCGA workgroup. Raw counts were normalized using RNA sequencing by expectation maximization. Level-3 gene quantification data were downloaded from the TCGA data portal and were further checked for quality.

Sample processing

Harvard [1]: Genomic DNA was extracted from fresh-frozen tissues using QIAmp tissue kit according to the manufacturer's instruction. DNA concentration was measured by DyNA Quant 200 fluorometer (Hoefer). Spain [2]: DNA was extracted from frozen specimens using a standard phenol chloroform extraction method. Sweden [3]: DNA was extracted from the biopsies using QIAamp DNA Mini Kit (Qiagen). Norway [4]: DNA was extracted from the snap-frozen lung adenocarcinoma tissue using Maxwell 16 DNA Purification kit (http://www.promega.com) and the Maxwell 16 instrument after standard procedure. Standard TRIZOL methods (Invitrogen, Carlsbad, CA, USA) were used to extract total RNA and the procedure was done according to manufacturer`s instruction. RNA quantity and quality (yield, 260/280 ratio and 260/230 ratio) were determined using the NanoDrop ND-1000 spectrometer (NanoDrop technologies) and RNA integrity numbers (RIN) were measured using the 2100 Bioanalyzer (Agilent technologies, Santa Clara, CA). TCGA [5]: TCGA Research Network utilized the Genome Characterization Pipeline to transform tissue samples into rich, publicly available data (https://www.cancer.gov/about-nci/organization/ccg).

Given the potential batch effect across different study centers, we applied the sample quality control pipeline, including batch effect correction, for samples with RNA expression and DNA methylation probes in each of center. Statistical simulation studies revealed that quantile normalization plus batch adjustment, which used in our study, was the best pipeline for correction of batch effect [6]. It well controls the both inter and intra variations, avoiding false positives caused by data bias.

REFERENCES

1. Suk Heist R, Zhou W, Cogan-Drew T, Liu G, Su L, Neuberg D, Lynch TJ, Wain JC, Christiani DC. MDM2 polymorphism and recurrence-free and overall survival in early stage non-small cell lung cancer (NSCLC). J Clin Oncol. 2006; 24:7221. https://doi.org/10.1200/jco.2005.23.16_suppl.9524 PMID: 27946733

2. Sandoval J, Mendez-Gonzalez J, Nadal E, Chen G, Carmona FJ, Sayols S, Moran S, Heyn H, Vizoso M, Gomez A, Sanchez-Cespedes M, Assenov Y, Müller F, et al. A prognostic DNA methylation signature for stage I non-small-cell lung cancer. J Clin Oncol. 2013; 31:4140–7. https://doi.org/10.1200/JCO.2012.48.5516 PMID: 24081945

3. Bjaanaes MM, Fleischer T, Halvorsen AR, Daunay A, Busato F, Solberg S, Jorgensen L, Kure E, Edvardsen H, Borresen-Dale AL, Brustugun OT, Tost J, Kristensen V, et al. Genome-wide DNA methylation analyses in lung adenocarcinomas: Association with EGFR, KRAS and TP53 mutation status, gene expression and prognosis. Mol Oncol. 2016; 10:330–43. https://doi.org/10.1016/j.molonc.2015.10.021 PMID: 26601720

4. Karlsson A, Jonsson M, Lauss M, Brunnstrom H, Jonsson P, Borg A, Jonsson G, Ringner M, Planck M, Staaf J. Genome-wide DNA methylation analysis of lung carcinoma reveals one neuroendocrine and four adenocarcinoma epitypes associated with patient outcome. Clin Cancer Res. 2014; 20:6127–40.

https://doi.org/10.1158/1078-0432.CCR-14-1087
PMID: 25278450

5. Weinstein JN, Collisson EA, Mills GB, Shaw KR, Ozenberger BA, Ellrott K, Shmulevich I, Sander C, Stuart JM. The Cancer Genome Atlas Pan-Cancer analysis project. Nat Genet. 2013; 45:1113–20.
https://doi.org/10.1038/ng.2764
PMID: 24071849

6. Sun Z, Chai HS, Wu Y, White WM, Donkena KV, Klein CJ, Garovic VD, Therneau TM, Kocher JP. Batch effect correction for genome-wide methylation data with Illumina Infinium platform. BMC Med Genomics. 2011; 4:84.
https://doi.org/10.1186/1755-8794-4-84
PMID:22171553
