# Supplementary material for: Trans-omics biomarker model improves prognostic prediction accuracy for early-stage lung adenocarcinoma
Source: Aging (Albany NY). 2019 Aug 21;11(16):6312–35. doi: 10.18632/aging.102189 (PMC6738411; doi:10.18632/aging.102189)
Supplement: Supplementary Figures [file aging-11-102189-s003.pdf]

SUPPLEMENTARY FIGURES

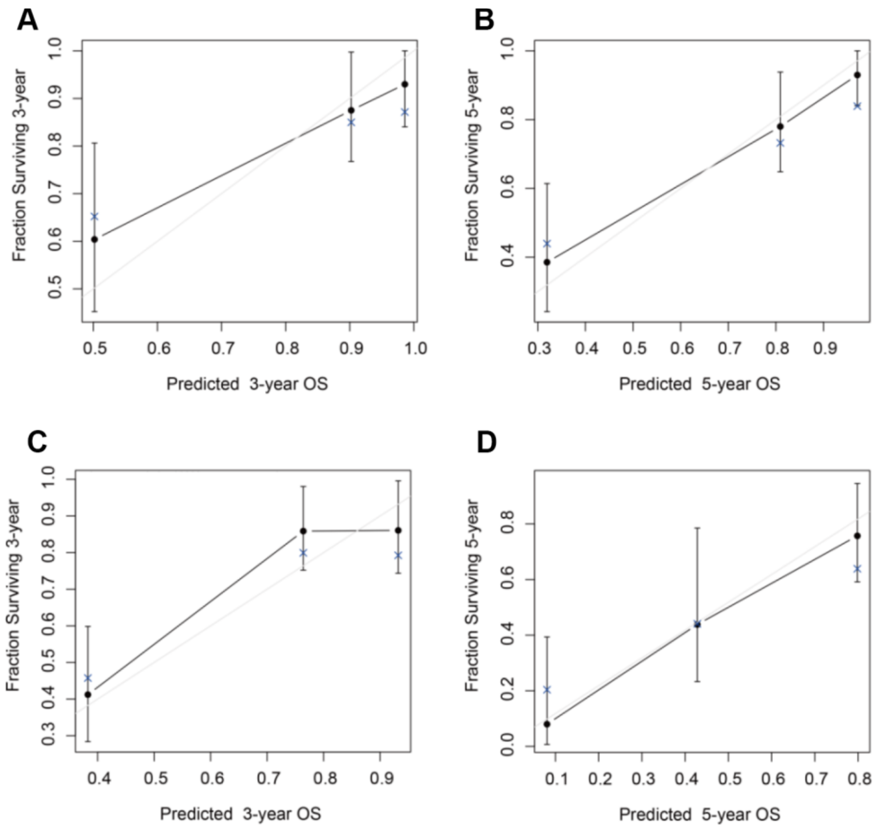

Supplementary Figure 1. Nomogram model calibration curves: (**A**) 3-year calibration curve in the discovery phase; (**B**) 5-year calibration curve in the discovery phase; (**C**) 3-year calibration curve in the validation phase; and (**D**) 5-year calibration curve in the validation phase. The x-axis shows nomogram-predicted probability of survival, and the y-axis shows actual survival as estimated by Kaplan-Meier. Gray line represents a perfect estimated outcome in an ideal model and perfect association with the actual outcome. Black line represents estimated outcome of the model, and closer alignment with the gray line represents better performance.

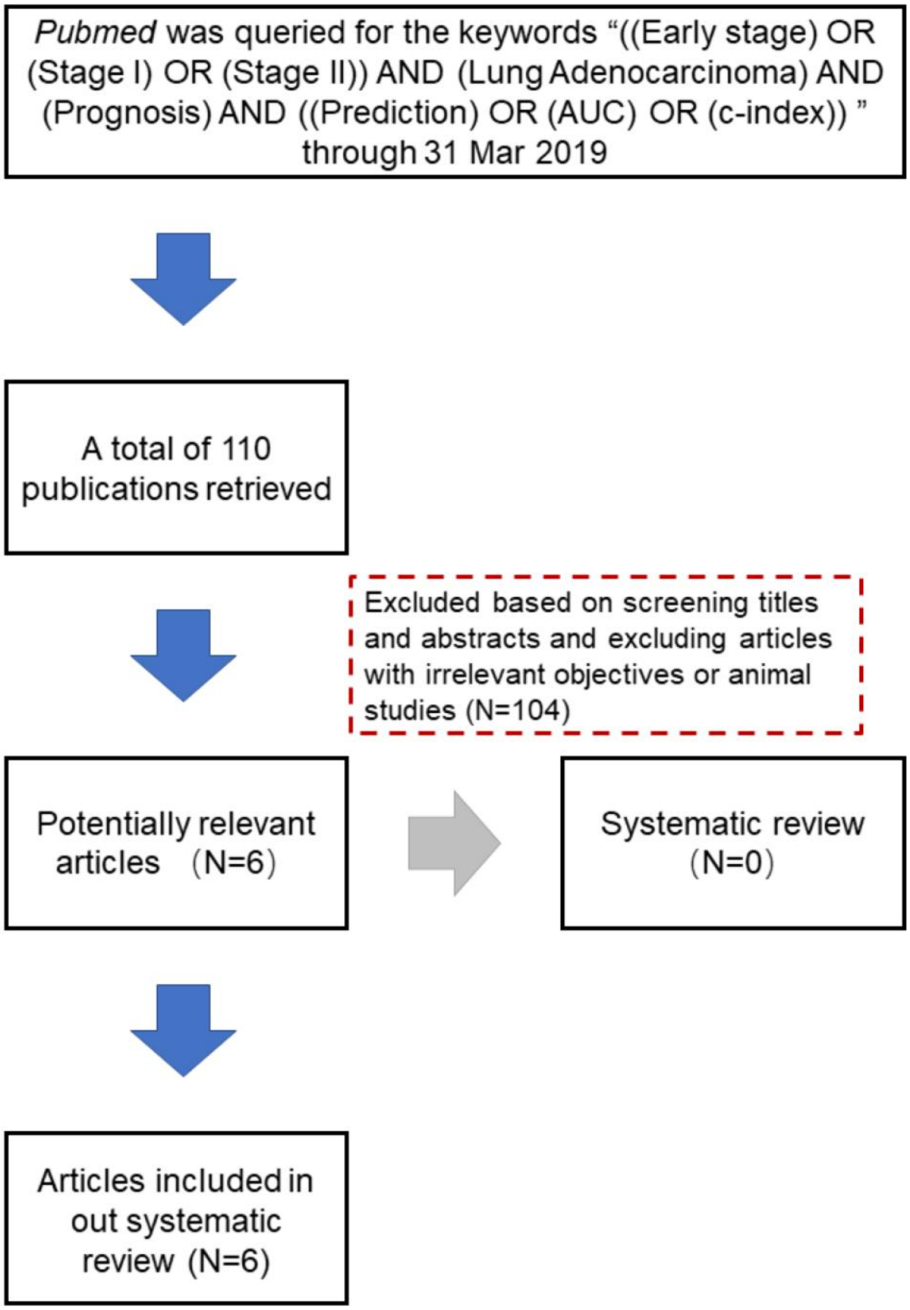

Supplementary Figure 2. Flowchart of systematic review.

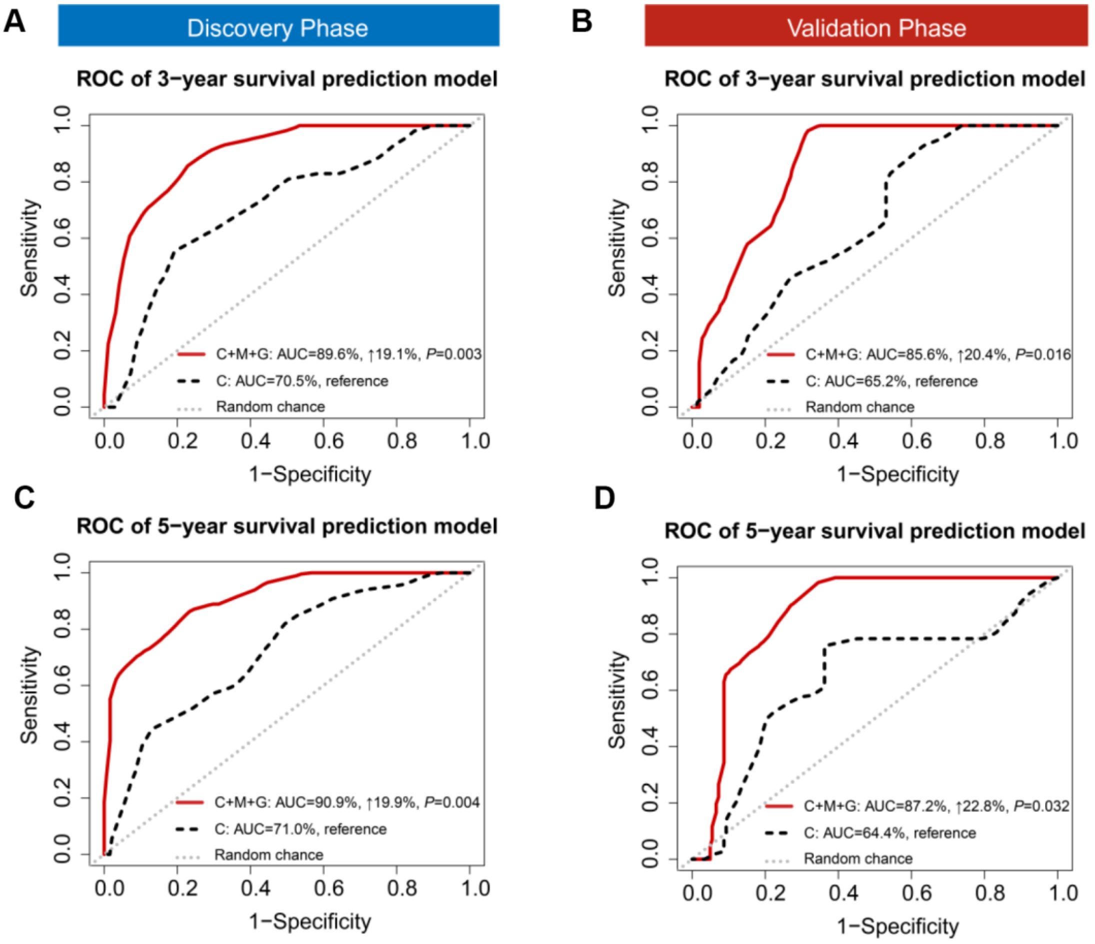

Supplementary Figure 3. The ROC curves of the prognostic model with chemotherapy information in sensitivity analysis. Time-dependent receiver operating characteristic (ROC). ROC was used to evaluate the performance of prognostic models for 3-year (**A**) and 5-year (**B**) overall survival prediction in the discovery phase. ROC also was used to evaluate the performance of prognostic models for 3-year (**C**) and 5-year (**D**) overall survival prediction in the validation phase. C: clinical model; C+M+G: clinical, DNA methylation, and gene expression model.

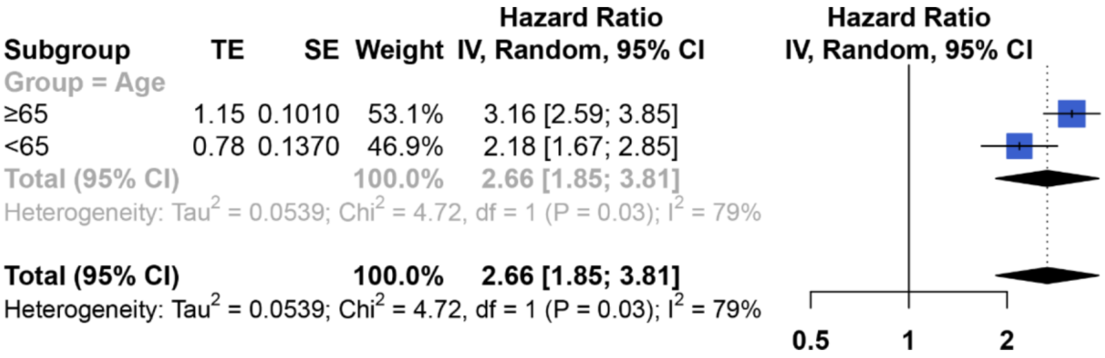

Supplementary Figure 4. Hazard ratio of trans-omics biomarker risk score in elderly and young groups.

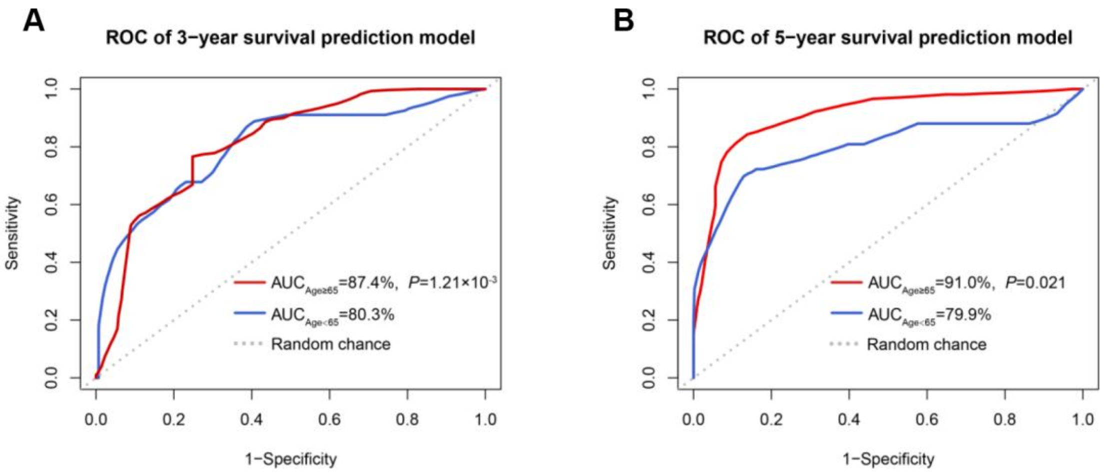

Supplementary Figure 5. Time-dependent ROC curves in elderly and young groups. ROC curve was used to evaluate the performance of prognostic models for 3-year (**A**) and 5-year (**B**) overall survival prediction.
