# Supplementary material for: Trans-omics biomarker model improves prognostic prediction accuracy for early-stage lung adenocarcinoma
Source: Aging (Albany NY). 2019 Aug 21;11(16):6312–35. doi: 10.18632/aging.102189 (PMC6738411; doi:10.18632/aging.102189)
Supplement: Supplementary Tables [file aging-11-102189-s002.pdf]

## SUPPLEMENTARY TABLES

**Supplementary Table 1. Early-stage LUAD prognosis-associated DNA methylation probes in discovery and validation phases.**

| Probe | Discovery phase | | | | | Validation phase | | | |
|---|---|---|---|---|---|---|---|---|---|
| | HR | 95% CI | | *P* | FDR-*q* | HR | 95% CI | | *P* |
| cg06835509 | 0.2 | 0.1 | 0.5 | 1.27E-04 | 1.54E-03 | 0.1 | 0.0 | 0.6 | 1.03E-02 |
| cg25702780 | 8.7 | 2.8 | 27.6 | 2.20E-04 | 1.54E-03 | 5.5 | 1.6 | 18.8 | 6.59E-03 |
| cg11123595 | 4.1 | 1.9 | 9.1 | 4.47E-04 | 1.68E-03 | 3.1 | 1.1 | 8.7 | 3.58E-02 |
| cg09916234 | 7.4 | 2.3 | 23.8 | 7.74E-04 | 2.32E-03 | 14.0 | 1.4 | 138.0 | 2.38E-02 |
| cg04101194 | 5.4 | 1.8 | 16.2 | 2.91E-03 | 7.28E-03 | 6.5 | 2.2 | 19.2 | 7.64E-04 |
| cg01431482 | 3.1 | 1.3 | 7.4 | 9.73E-03 | 1.37E-02 | 6.8 | 1.3 | 36.1 | 2.55E-02 |
| cg05944877 | 0.4 | 0.2 | 0.8 | 7.69E-03 | 1.37E-02 | 0.2 | 0.1 | 0.8 | 2.37E-02 |
| cg19196826 | 7.1 | 1.7 | 30.6 | 8.22E-03 | 1.37E-02 | 9.5 | 2.0 | 45.6 | 4.74E-03 |
| cg20149022 | 4.7 | 1.5 | 14.2 | 6.49E-03 | 1.37E-02 | 16.8 | 1.4 | 197.0 | 2.46E-02 |
| cg22122862 | 3.4 | 1.3 | 8.5 | 1.00E-02 | 1.37E-02 | 5.4 | 1.0 | 29.9 | 5.13E-02 |
| cg25947773 | 2.7 | 1.1 | 6.4 | 2.44E-02 | 3.05E-02 | 18.1 | 2.5 | 129.0 | 3.87E-03 |
| cg23780635 | 2.6 | 1.1 | 6.5 | 3.66E-02 | 4.53E-02 | 16.6 | 1.7 | 168.0 | 1.70E-02 |

Hazard ratios (HR), 95% confidence intervals (95% CI), and *P*-values were derived from Cox regression model adjusted for age, gender, clinical stage, and smoking status.
LUAD, lung adenocarcinoma; FDR, false discovery rate

**Supplementary Table 2. Early-stage LUAD prognosis-associated gene expression probes in discovery and validation phases.**

| Gene | Discovery phase | | | | | Validation phase | | | |
|---|---|---|---|---|---|---|---|---|---|
| | HR | 95% CI | | *P* | FDR-*q* | HR | 95% CI | | *P* |
| *BLM* | 2.6 | 1.4 | 5.1 | 4.11E-03 | 7.19E-03 | 1.4 | 1.1 | 1.9 | 1.02E-02 |
| *CASC5* | 2.7 | 1.5 | 4.9 | 1.43E-03 | 6.14E-03 | 1.3 | 1.1 | 1.6 | 3.21E-03 |
| *FHIT* | 0.2 | 0.1 | 0.8 | 2.01E-02 | 2.01E-02 | 0.6 | 0.4 | 0.7 | 5.17E-05 |
| *GMPS* | 4.1 | 1.7 | 10.0 | 1.75E-03 | 6.14E-03 | 1.9 | 1.3 | 3.0 | 3.08E-03 |
| *MSH2* | 4.3 | 1.6 | 11.4 | 4.04E-03 | 7.19E-03 | 1.5 | 1.0 | 2.2 | 3.13E-02 |
| *SLC34A2* | 0.5 | 0.2 | 0.8 | 1.23E-02 | 1.44E-02 | 0.8 | 0.7 | 0.9 | 1.58E-03 |
| *ZNF429* | 0.1 | 0.0 | 0.6 | 1.08E-02 | 1.44E-02 | 0.7 | 0.5 | 0.9 | 1.53E-02 |

Hazard ratios (HR), 95% confidence intervals (95% CI), and *P*-values were derived from Cox regression model adjusted for age, gender, clinical stage, and smoking status.
LUAD, lung adenocarcinoma; FDR, false discovery rate

**Supplementary Table 3. Potential DNA methylation–gene expression pathways associated with early-stage LUAD prognosis in causal mediation analysis.**

| DNA methylation–gene expression | Discovery phase | | | | | | Validation phase | | | | | |
|---|---|---|---|---|---|---|---|---|---|---|---|---|
| | $HR_{DE}$ | 95% $CI_{DE}$ | $P_{DE}$ | $HR_{IDE}$ | 95% $CI_{IDE}$ | $P_{IDE}$ | $HR_{DE}$ | 95% $CI_{DE}$ | $P_{DE}$ | $HR_{IDE}$ | 95% $CI_{IDE}$ | $P_{IDE}$ |
| cg25947773-*GMPS* | 1.9 | 0.2–19.6 | 6.03E-01 | 3.3 | 1.3–8.6 | 1.52E-02 | 18.1 | 2.5–129 | 3.87E-03 | 2.9 | 1.4–6.0 | 5.77E-03 |
| cg25947773-*BLM* | 1.9 | 0.2–19.6 | 6.03E-01 | 2.7 | 1.2–6.2 | 2.08E-02 | 18.1 | 2.5–129 | 3.87E-03 | 2.2 | 1.2–4.3 | 1.57E-02 |
| cg05944877-*MSH2* | 0.6 | 0.1–5.2 | 6.76E-01 | 0.3 | 0.1–0.9 | 2.91E-02 | 0.2 | 0.1–0.8 | 2.37E-02 | 0.7 | 0.5–1.0 | 4.92E-02 |
| cg23780635-*BLM* | 3.6 | 0.3–44.2 | 3.23E-01 | 2.5 | 1.1–6.0 | 3.65E-02 | 16.6 | 1.7–167.6 | 1.70E-02 | 2.3 | 1.2–4.4 | 1.77E-02 |
| cg23780635-*GMPS* | 3.6 | 0.3–44.2 | 3.23E-01 | 2.6 | 1.0–6.7 | 4.73E-02 | 16.6 | 1.7–167.6 | 1.70E-02 | 2.8 | 1.3–5.8 | 7.43E-03 |
| cg23780635-*MSH2* | 3.6 | 0.3–44.2 | 3.23E-01 | 2.7 | 1.0–7.5 | 4.83E-02 | 16.6 | 1.7–167.6 | 1.70E-02 | 1.7 | 1.0–2.8 | 4.78E-02 |

Sobel method was used for calculations, with adjustment for age, gender, clinical stage, and smoking status.
$HR_{DE}$, 95% $CI_{DE}$, and $P_{DE}$ evaluated direct effects of DNA methylation on lung adenocarcinoma (LUAD) survival.
$HR_{IDE}$, 95% $CI_{IDE}$, and $P_{IDE}$ evaluated indirect effects of DNA methylation on LUAD survival, mediated through corresponding gene expression.

**Supplementary Table 4. Systematic review of accuracy of early-stage LUAD prognostic model.**

| PMID | Author | Year | Stage | *N* | Validation | Outcome | Statistical model | Predictor | AUC/c-index |
|---|---|---|---|---|---|---|---|---|---|
| 28122330 | Chen M | 2017 | I, II | 830 | Yes | 5-year survival | Cox proportional hazards model | 7 lnc-RNA | 0.65 |
| 28922552 | Qi L | 2017 | I | 542 | Yes | 5-year recurrence | Cox proportional hazards model | 9 mRNA | 0.55 |
| 29756233 | Martínez-Terroba E | 2018 | I, II | 353 | Yes | 5-year survival | Cox proportional hazards model | 12 protein | 0.65 |
| 24046125 | Kim DH | 2014 | I | 102 | No | Recurrence after Curative surgical Resection | Cox proportional hazards model | F-18 fluoro-2-deoxyglucose Positron emission tomography | 0.69 |
| 27524912 | Sun Y | 2016 | I | 92 | No | First 7.5-year survival | Cox proportional hazards model | 2 mRNA | 0.75 |
| 26947549 | Okayama A | 2016 | I | 59 | No | 5-year recurrence | Multiple reaction monitoring | phospho-SSFA2 | 0.79 |

LUAD, lung adenocarcinoma

**Supplementary Table 5. DNA methylation information: 450K bead array from ENCODE/HAIB.**

| DNA methylation probes | Chromosome | Band | Base position | Gene | Name | Region |
|---|---|---|---|---|---|---|
| cg01431482 | 1 | 1p36.32 | 2989086 | *PRDM16* | PR domain containing 16 | 1-intron |
| cg22122862 | 1 | 1p36.32 | 2987915 | *PRDM16* | PR domain containing 16 | 1-intron |
| cg25947773 | 2 | 2q36.1 | 223771011 | *ACSL3* | Acyl-CoA synthetase long-chain family member 3 | 3-intron |
| cg25702780 | 3 | 3q26.2 | 169376299 | *MECOM* | MDS1 and EVI1 complex locus | 1-intron |
| cg11123595 | 3 | 3q26.2 | 169376619 | *MECOM* | MDS1 and EVI1 complex locus | 1-intron |
| cg09916234 | 4 | 4p16.3 | 1976220 | *WHSC1* | Wolf-Hirschhorn syndrome candidate 1 | 18-intron |
| cg04101194 | 4 | 4p15.2 | 25656866 | *SLC34A2* | Solute carrier family 34 (sodium phosphate), member 2 | 5'-UTR |
| cg19196826 | 7 | 7p22.2 | 3018392 | *CARD11* | Caspase recruitment domain family, member 11 | 1-intron |
| cg23780635 | 8 | 8p23.3 | 1880114 | *ARHGEF10* | Rho guanine nucleotide exchange factor (GEF) 10 | 15-intron |
| cg20149022 | 8 | 8p23.3 | 1863072 | *ARHGEF10* | Rho guanine nucleotide exchange factor (GEF) 10 | 9-intron |
| cg06835509 | 16 | 16p12.2 | 23939098 | *PRKCB* | Protein kinase C, beta | 2-intron |
| cg05944877 | 16 | 16p12.2 | 24197864 | *PRKCB* | Protein kinase C, beta | 15-intron |

**Supplementary Table 6. Interaction analysis (biomarkers *vs.* age) for overall survival.**

| Interaction term with age | Discovery phase | | | | Validation phase | | | |
|---|---|---|---|---|---|---|---|---|
| | HR | CL | CU | P value | HR | CL | CU | P value |
| cg01431482 | 0.93 | 0.73 | 1.19 | 5.89E-01 | 1.05 | 0.87 | 1.27 | 5.84E-01 |
| cg22122862 | 1.01 | 0.77 | 1.33 | 9.46E-01 | 1.14 | 0.96 | 1.36 | 1.44E-01 |
| cg25947773 | 1.15 | 0.91 | 1.46 | 2.45E-01 | 1.05 | 0.87 | 1.27 | 6.19E-01 |
| cg11123595 | 0.86 | 0.65 | 1.16 | 3.28E-01 | 1.03 | 0.90 | 1.18 | 6.57E-01 |
| cg25702780 | 0.95 | 0.66 | 1.35 | 7.58E-01 | 0.96 | 0.80 | 1.15 | 6.35E-01 |
| cg04101194 | 0.90 | 0.58 | 1.39 | 6.42E-01 | 1.03 | 0.90 | 1.19 | 6.37E-01 |
| cg09916234 | 1.04 | 0.64 | 1.68 | 8.77E-01 | 1.00 | 0.77 | 1.30 | 9.91E-01 |
| cg19196826 | 0.99 | 0.77 | 1.29 | 9.60E-01 | 0.89 | 0.71 | 1.10 | 2.79E-01 |
| cg20149022 | 0.90 | 0.57 | 1.41 | 6.40E-01 | 1.00 | 0.76 | 1.33 | 9.75E-01 |
| cg23780635 | 1.07 | 0.81 | 1.41 | 6.43E-01 | 1.24 | 1.00 | 1.54 | 5.12E-02 |
| cg05944877 | 0.95 | 0.72 | 1.26 | 7.26E-01 | 0.95 | 0.84 | 1.08 | 4.63E-01 |
| cg06835509 | 0.89 | 0.68 | 1.16 | 3.79E-01 | 0.86 | 0.72 | 1.03 | 1.11E-01 |
| SLC34A2 | 1.01 | 0.94 | 1.08 | 7.62E-01 | 1.00 | 0.97 | 1.02 | 7.43E-01 |
| ACSL3 | 1.17 | 1.01 | 1.35 | 3.62E-02 | 0.99 | 0.97 | 1.02 | 5.76E-01 |
| ARHGEF10 | 0.85 | 0.76 | 0.96 | 6.48E-03 | 1.00 | 0.98 | 1.01 | 4.71E-01 |
| WHSC1 | 1.16 | 1.01 | 1.33 | 3.95E-02 | 1.03 | 0.99 | 1.07 | 1.56E-01 |
| MECOM | 1.01 | 0.94 | 1.09 | 7.55E-01 | 1.00 | 0.97 | 1.04 | 9.16E-01 |
| PRKCB | 0.98 | 0.90 | 1.06 | 5.62E-01 | 1.00 | 0.97 | 1.04 | 8.22E-01 |
